# Optical genome mapping identifies hidden structural variation in acute myeloid leukemia: Two case reports

**DOI:** 10.1016/j.htct.2024.06.011

**Published:** 2024-11-08

**Authors:** Harmanpreet Singh, Nikhil Shri Sahajpal, Vivek Gupta, Jaspreet Farmaha, Ashutosh Vashisht, Ashis K Mondal, Ravindra Kolhe

**Affiliations:** a Medical College of Georgia, Augusta University, GA, USA; b Greenwood Genetic Center, Greenwood, SC, USA; c Department of Pathology, Government Institute of Medical Sciences, Noida, India

## Introduction

Acute myeloid leukemia (AML) is a cancer of blood and bone marrow characterized by a higher percentage of immature or undifferentiated myeloid precursor cells known as myeloblasts or blasts. It is the most common leukemia found in older adults accounting for about 80 % of all cases. Genetic abnormalities defining AML primarily include t(8;21)(q22;q22.1)/*RUNX1::RUNX1T1* (12 %), the *NPM1* mutation (25–30 %), isocitrate dehydrogenase (IDH) mutations (15–20 %), the *FLT3-ITD* mutation (40 %), t(9;11)(p21.3;q23.3)/*MLLT3::KMT2A*, inv(16)(p13.1q22) or t(16;16)(p13.1;q22)/*CBFB::MYH11*, t(6;9)(p23;q24)/ *DEK::NUP214*, and inv(3)(q21.3q26.2).^1^ Of these rearrangements, aberrations involving *KMT2A* (11p23) have been reported in pediatric and adult AML^2^ with 90 different fusion partners identified till now.^3^ Among the fusion partners *MLLT10* (10p12.31) has been reported as the most common fusion partner in AML.^4^

Similarly, the *RUNX1* gene is involved in >50 chromosome translocations of *de novo* and therapy-related AML.^5^ The most common translocations involving *RUNX1* include t(12;21) observed in 25 % of pediatric acute lymphoblastic leukemia (ALL), t(8; 21) in 10 % of adult AML, and t(3;21) in therapy-related AML and myelodysplastic syndrome (MDS). Amongst the rare translocations involving *RUNX1* is t(16; 21)(q24;q22), which results in a fusion with the *CBFA2T3* gene, observed in AML and MDS.^6^ Johanna et al*.* reported 19 cases of adult AML involving t(16; 21)(q24;q22): four cases demonstrated *de novo* AML at diagnosis, whereas the other patients had previously undergone chemotherapy for different tumors or lymphomas.^7^

Currently, the genetic workup of AML relies on fluorescence in situ hybridization (FISH), karyotyping and targeted next-generation sequencing panels. We hereby present two cases of AML, in which the standard of care (SOC) cytogenetic and molecular analysis resulted in negative findings, but the presence of structural variants (SVs) associated with AML were revealed using optical genome mapping (OGM) analysis.

### Case report 1

The sample of a 30-year-old female with no previous known clinical history was received in the pathology laboratory for molecular workup. Bone marrow examinations (aspiration smears, clot section, touch preparation, and core biopsy) were done. The complete blood count was white blood cell count (WBC): 3.7 × 10^3^/µL, hemoglobin (Hb): 8.8 g/µL, hematocrit (Hct): 25.3 %, platelet count: 73×10^3^/µL. The specimen was submitted to molecular investigations.

Bone marrow was hypercellular for age with the cellular profile being consistent with AML M1 (French-American-British - FAB classification) with blasts comprising 81 %. The karyotype was 46, XX - negative for any aberration. FISH testing was performed with the following probes: t(9;22) *BCR::ABL1/ASS1*, t(15;17) *PML::RARA*), t(8;21) *RUNXIT1::RUNX1*, 8p11 *FGFRI* rearrangement, inv(3) *MECOM* rearrangement, i(17q), 17q *RARA* rearrangement, del(5q) *EGR1*, 11q23 *KMT2A* (*MLL*), del(7q)/monosomy7, trisomy 8, del(20q), 11p15.4 *NUP98* rearrangement, inv(16), t(16;16) *CBFB* rearrangement, 12p13 *ETV6* rearrangement, 4q12 *FIP* *1L1/CHIC2/PDGFRA*, and the 5q32 *PDGFRB* rearrangement was also found to be negative.

Amplicon-based targeted next generation sequencing (NGS) was performed using a myeloid panel for the following genes: *ABL1, BRAF, CBL, CSF3R, DNMT3A, FLT3, GATA2, HRAS, IDH1, IDH2, JAK2, KIT, KRAS, MPL, MYD88, NPM1, NRAS, PTPN11, SETBP1, SF3B1, SRSF2, UZAF1*, and *WT1*. All of which were found negative for any mutation. Moreover, evaluations of the full genes (*ASXL1, BCOR, CALR, CDKNZA, CEBPA, ETV6, EZH2, FBXW7, IKZF1, NFI, PHF6, PRPF8, PTEN, RB1, RUNX1, SH2B3, STAG2, TET2, TP53*, and *ZRSR2*) also failed to detect any pathogenic alteration.

This sample was received in the pathology laboratory at Augusta University to perform OGM after performing the SOC testing. OGM was performed as per the manufacturer's protocols (Bionano Genomics Inc., San Diego, CA). Briefly, ultra-high-molecular-weight DNA from the bone marrow sample was isolated, labelled, and processed for analysis on the Bionano Genomics Saphyr platform; for more details on the procedure refer to Sahajpal et al.^8^ Genome analysis was performed using the rare variant pipeline included in the Bionano Access v.1.7.2/ Bionano Solve (v.3.7.2) software.

**Figure 1:**
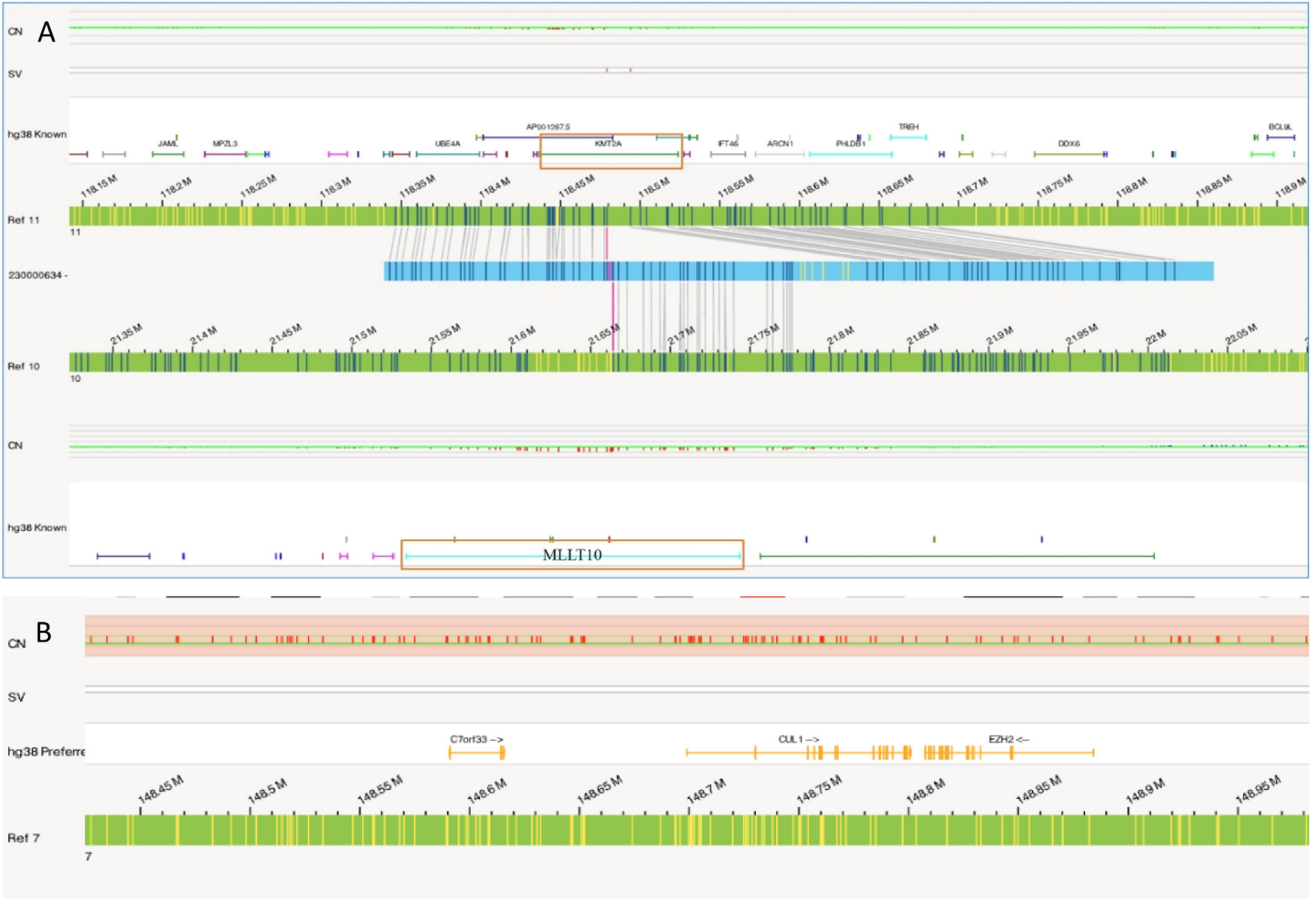
Structural variant view in which optical genome mapping in Case 1 reveals; a) gene fusions detected with translocations between chromosomes 10 and 11: KMT2A:MLLT10, b) Deletion of the *EZH2* gene in chromosome 7.

**Figure 2:**
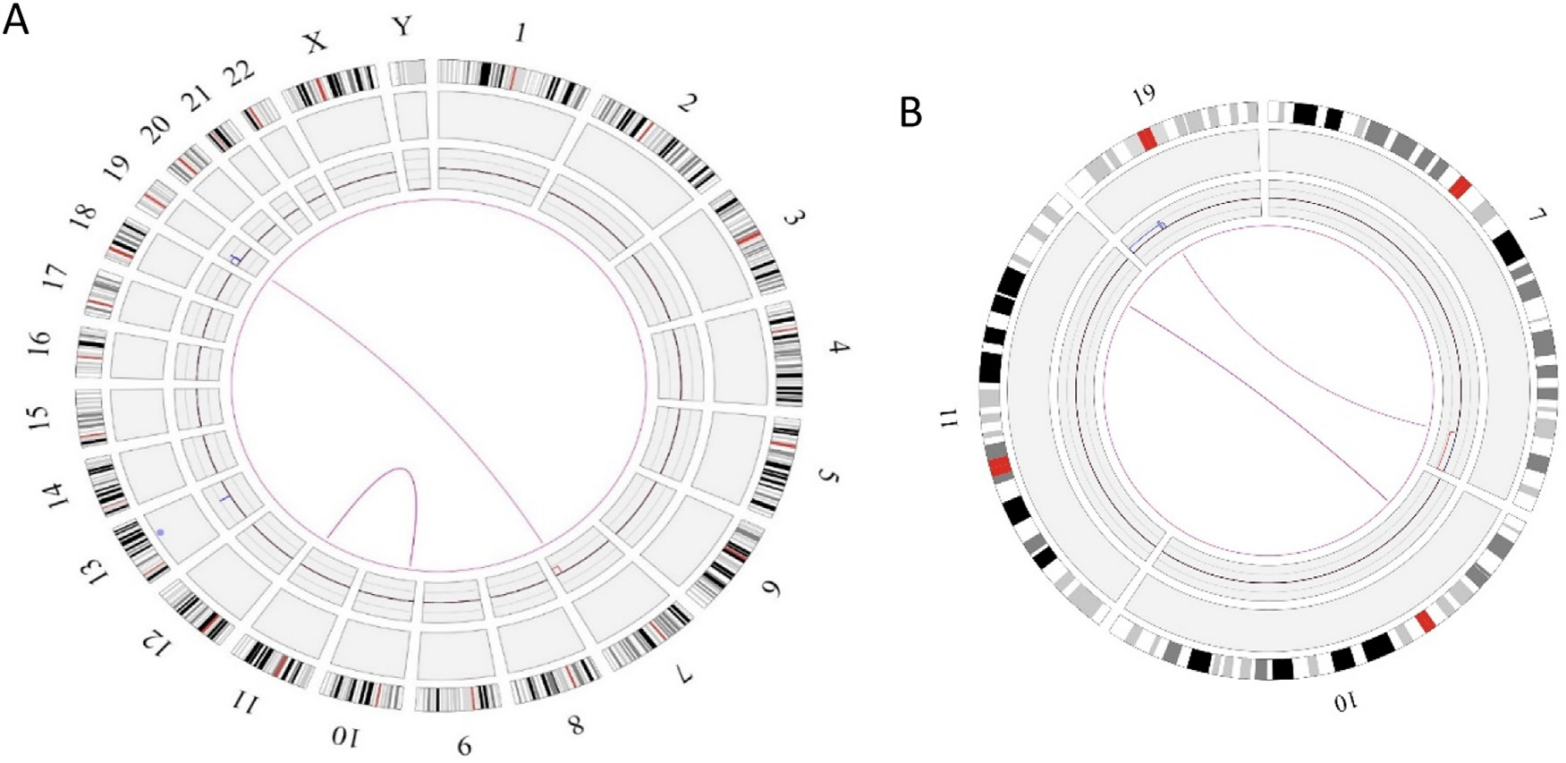
Circos plot showing the structural variants (SVs) in Case 1. a) Optical genome mapping helped to find the clinically relevant unbalanced translocation event between chromosomes 7 and 19, which resulted in copy number variation loss in chromosome 7 and a balanced rearrangement event between chromosomes 10 and 11. b) Enlarged SV view of the chromosome that has relevant SVs.

**Figure 3:**
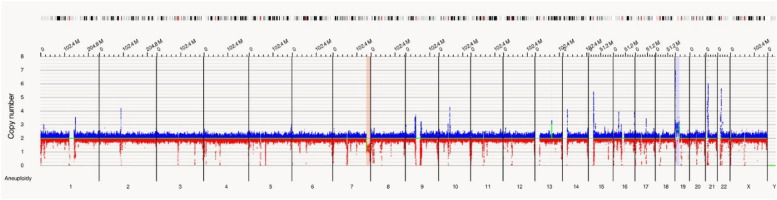
Whole genome view of Case 1 showing the copy number variations for chromosome 7 (loss in copy number) and chromosome 19 (gain in copy number).

In this case OGM identified two SVs, an unbalanced translocation t(7;19)(q34;p13.11)(142,520,860;17,398,836), resulting in one copy deletion of the *EZH2* gene as shown in Figure 1. More importantly, a complex balanced rearrangement, ins(10;11) (p12.3;11q23) (21,664,036;118,479,068) was found that resulted in the *KMT2A::MLLT10* fusion as shown in Figure 1. Figure 2a illustrates the circos plot showing the SV events i.e. interchromosomal translocations between chromosomes 10 and 11, and between chromosomes 7 and 19. Figure 2b shows the enlarged SV view of the chromosome that has relevant SVs in this case. Figure 3 shows the whole genome view with copy number variations (CNVs) on chromosomes 7 and 19 in this case.

### Case report 2

A 41-year-old male presented with a history of diffuse large B cell lymphoma (DLBCL). The hematological workup for complete blood count showed WBC: 4.4 × 10^3^/µL, Hb: 11.4 g/µL, Hct: 33.2 % and platelet count: 58 × 10^3^/µL. Bone marrow examinations (aspiration smears, clot section, touch preparation, and core biopsy) were done. The bone marrow aspirate was also submitted to molecular investigations.

The bone marrow was normocellular (60 % cellular), the myeloid to erythroid (M:E) ratio was 2.1 and blasts comprised 11 %. The conclusive findings of the bone marrow examination showed no overt evidence of involvement of any lymphoproliferative disorder. Cytological atypia was seen in all three cell lines. Immunohistochemistry was positive for CD34. Flow cytometry identified the unique myeloblast population. Overall findings showed suspicion of a myeloid neoplasm. Karyotyping was 46 XY - negative for any aberration. FISH testing using the probes: t(9;22) *BCR::ABL1/ASS1*, 8p11 *FGFR1* rearrangement, del(5q) *EGR1*, del(7q)/monosomy7, 11q23 *KMT2A (MLL),* trisomy 8, inv(16), t(16;16) *CBFB* rearrangement, del(20q), 12p13 *ETV6* rearrangement, 4q12 *FIP* *1L1/CHIC2/PDGFRA*, 5q32 *PDGFRB* rearrangement, and 11p15.4 *NUP98* rearrangement was also found to be negative. Amplicon-based targeted NGS with the myeloid panel used in Case 1 was also employed in this case, but no pathogenic alterations were identified in any gene.

**Figure 4:**
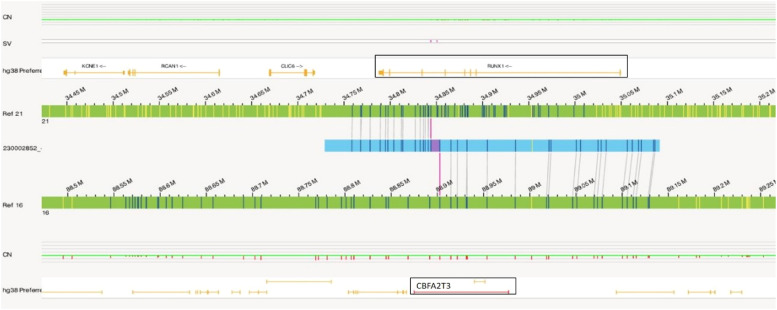
Showing the structural variant view in which optical genome mapping in Case 2 identified the translocation between chromosomes 21 and 16 resulting in the *CBFA2T3::RUNX1* gene fusion.

**Figure 5:**
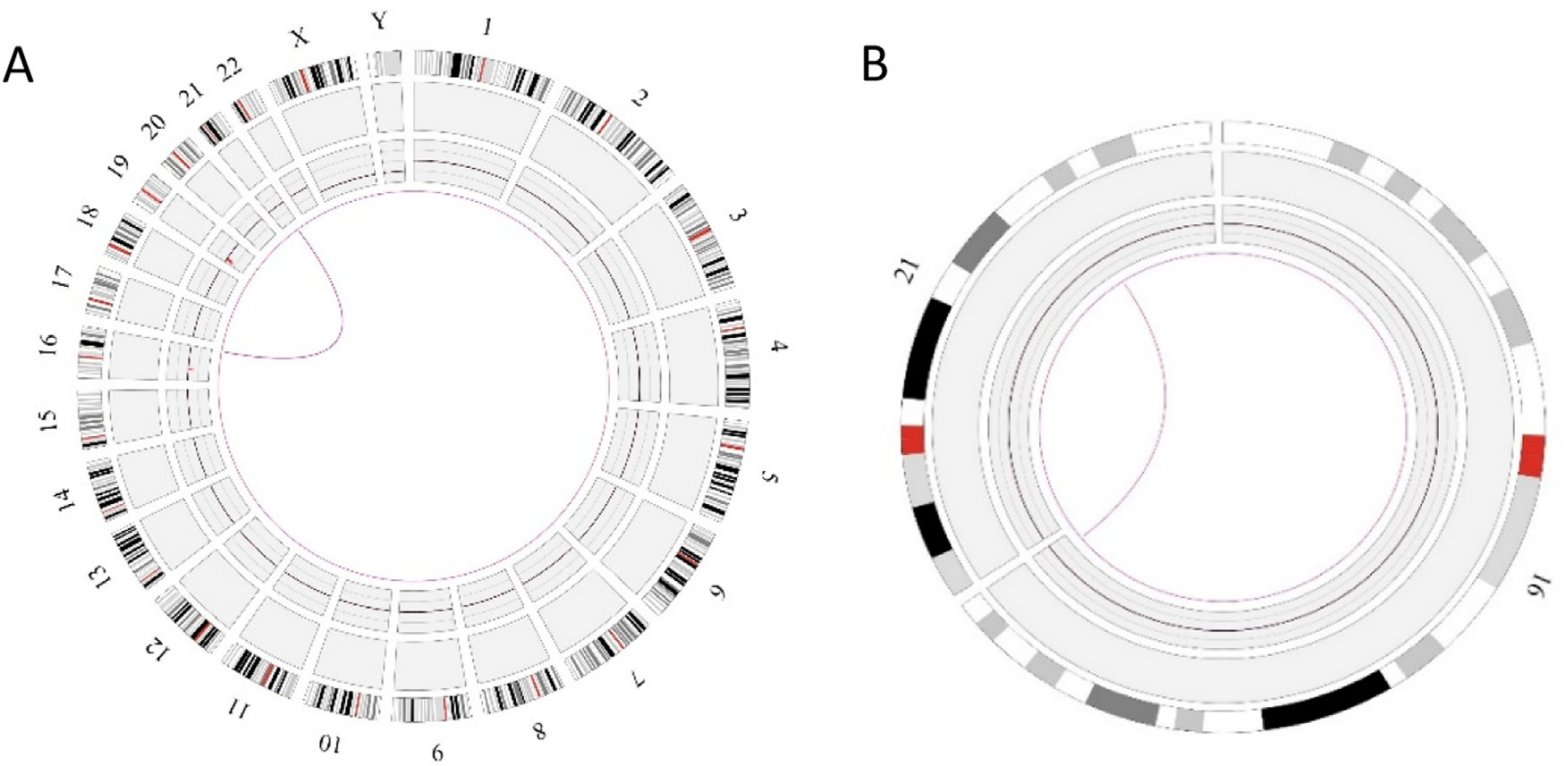
Circos plot summarizing the structural variant event in Case 2 a) the interchromosomal translocation between chromosomes 16 and 21, b) enlarged structural variant (SV) view of the chromosome with relevant SVs.

Similar to Case 1, OGM analysis helped to identify a balanced translocation t(16;21)(q24.3;q22.12) (88,902,697;34,843,977), which resulted in the fusion of the *CBFA2T3::RUNX1* genes as shown in Figure 4. Figure 5a illustrates the circos plot which shows the translocation event between chromosomes 16 and 21, and Figure 5b shows the enlarged SV view of the chromosome having relevant SV.

### Interpretation and relevance of molecular findings

In Case 1, OGM unveiled a novel rearrangement of *KMT2A*(11p23), resulting in the *KMT2A::MLLT10* fusion. Although the World Health Organization (WHO) 2017 guidelines only included *MLLT3::KMT2A* t(9;11)(p21.3;q23) as an AML-specific gene fusion, there are now over 90 different *KMT2A* fusion partners listed in the 2022 WHO guidelines, with recommendation to label any *KMT2A* rearrangement as an AML defining aberration. Rearrangement of *KMT2A*, (previously known as the *MLL* gene), is seen in 4–5 % of *de novo* adult AML patients and up to 22 % in pediatric patients.^9^ The *KMT2A* rearrangement leads to the formation of a distinct oncogenic fusion protein affecting several downstream molecular signaling pathways.^10^ In addition to the *KMT2A::MLLT10* rearrangement, an unbalanced translocation t(7;19)(q34;p13.11) resulted in the deletion of the *EZH2* gene. *EZH2* belongs to the polycomb group (PcG) protein complex, located at 7q36.1, which promotes gene silencing by histone modification of H3 lysine 27 via methylation. Loss of *EZH2* function results in upregulation of *EZH2* target genes responsible for augmentation of proliferation and apoptosis invasions. Loss of the *EZH2* protein expression has been found in 50 % of individuals affected with AML and pathogenic variants of *EZH2* were found in 31.27 %.^11^^,^^12^

The t(16;21)(q24;q22), a fusion product of *RUNX1* with *CBFA2T3*, identified in Case 2 is a less common karyotypic event found in AML. *RUNX1* is part of the core binding factor transcription complex that regulates genes involved in hematopoietic development and differentiation. *CBFA2T3* also known as myeloid translocation gene (*MTG16* or *ETO2*) functions as a transcriptional corepressor in hematopoiesis. Hence, the oncogenic translocation results in a fusion protein that suppresses the expression of *RUNX1* target genes involved in hematopoietic differentiation, cell cycle regulation, and transforming growth factor β signaling pathways resulting in tumorigenic properties of the cells.^13^^,^^14^

## Discussion

We present two cases of AML that were labeled ‘normal’ as per the conventional SOC techniques. The application of the OGM technique helped in unravelling the pathogenic chromosomal abnormalities, underscoring the unique advantage of adopting OGM for the clinical diagnosis of AML samples.

OGM, a relatively recent molecular genomics technique, utilizes ultra-high molecular weight DNA molecules labelled at specific sequence motifs (CTTAAG) to generate 400x coverage or more, which allows the detection of even low-level mosaicism. This enables an unbiased assessment of genome-wide complex rearrangements and different classes of SVs (inversions, translocations, insertions, deletions¸ duplications) down to 500 base pairs (bp) in size. With its better resolution, OGM far exceeds the resolution of current SOC including karyotyping. With the resolution limit of 5 Mb, nearly 50 % of patients with *de novo* AML have normal karyotypes,^15^ as exemplified in the two case reports here.

NGS requires a larger panel to include all the structural variants with the utility and success being dependent upon the technique employed such as the amplicon or capture-based, short-read or long-read sequencing approaches. The amplicon-based technique using a myeloid panel with a read length of 200 bp was employed for these two case reports. However, this approach lacks the ability to detect larger deletions and insertions or CNVs, leading to a failure to detect the CNV event involving *EZH2*. Moreover, the approach is also limited in identifying translocations and gene fusions.

Finally, FISH, though useful, is a targeted approach that only uses probes of known or suspected SVs; it is not capable of discovering novel or additional SVs. Furthermore, depending upon the probe utilized, multiple attempts are needed thereby delaying the start of personalized therapy to achieve the best patient outcome. Moreover, breakpoint probes in FISH fail to identify fusion partners, which could be critical in prognosis and eventually disease outcome.^16^ As exemplified here, the 11q23 *KMT2A(MLL)* breakpoint FISH probe for AML used as recommended by the WHO, did not detect the fusion partners in Case 1, thereby limiting its clinical utility. The del (7q) probe recommended for AML, targets the 7q22 and 7q31.2 regions, and does not span *EZH2* which is located at 7q36.1.^17^ Consequently, the *EZH2* deletion was not detected using this probe.

In recent publications, OGM has demonstrated up to 100 % clinical concordance with traditional cytogenetic analysis and identified the additional clinically relevant SVs that remain beyond the scope of current SOC technologies**.**^18^^,^^19^ OGM provides a comprehensive ‘picture’ of the whole genome, enabling detailed analysis of SVs, unraveling complex or cryptic rearrangements and identifying translocated partners, all within a turnaround time of 3–4 days. Additionally, it helps to better classify the AML as per the FAB or European Leukemia Net (ELN) classifications and for therapeutic decisions for AML patients.

## Conclusion

These cases demonstrate the clinical utility of OGM, where a genome that was considered molecularly ‘normal’ was found to harbor genetic aberrations, including *KMT2A* and *RUNX1* rearrangements, which are recognized as AML-defining genetic abnormalities. Overall, this report supports the use of OGM as a next-generation cytogenomic tool in the routine diagnostic workflow.

## Conflicts of interest

The authors declare no conflicts of interest.
